# Association between Functional Polymorphisms of Foxp3 Gene and the Occurrence of Unexplained Recurrent Spontaneous Abortion in a Chinese Han Population

**DOI:** 10.1155/2012/896458

**Published:** 2011-07-13

**Authors:** Zaigui Wu, Zeshan You, Cai Zhang, Zhuyu Li, Xiumei Su, Xiuming Zhang, Yinguang Li

**Affiliations:** ^1^Department of Obstetrics and Gynecology, the First Affiliated Hospital of Medical College, Sun Yat-Sen University, Guangzhou 510080, Guangdong, China; ^2^Department of Obstetrics and Gynecology, People's Hospital of Chaozhou city, Chaozhou 515600, Guangdong, China; ^3^Center of Stem Cell Biology and Tissue Engineering, Sun Yat-sen University, Guangzhou 510080, Guangdong, China

## Abstract

Unexplained recurrent spontaneous abortion (URSA) is an alloimmune disease associated with the failure of fetal-maternal immunologic tolerance in which the regulatory T lymphocytes (Treg) play a pivotal role. It is well known that Forkhead box P3 (Foxp3) is a crucial regulatory factor for the development and function of Treg cells. It has also been established that deficiency of the Foxp3 gene suppresses the regulatory function of Treg cells. To determine if functional polymorphisms at the Foxp3 loci are associated with URSA in humans, we genotyped four common polymorphisms of Foxp3 gene in 146 unrelated URSA patients and 112 healthy women. The results showed that rs3761548A/C and rs2232365A/G polymorphisms were significantly associated with URSA. Additionally, we found that the allelic distribution of rs5902434 del/ATT in URSA group was slightly different from that in the control group. We conclude that functional polymorphisms of the Foxp3 gene may confer an important susceptibility to URSA in the Chinese Han population, probably by altering Foxp3 function and/or its expression.

## 1. Introduction

Recurrent spontaneous abortion (RSA), which is defined as two or more consecutive pregnancy losses before the 20th week of gestation from the last menstrual period, occurs in approximately 1% to 5% of women at reproductive age [1, 2]. Although many known causes of RSA including anatomic (15%), infectious (1%–2%), hormonal (20%), immunological (20%), and genetic (2%–5%) have been identified, a significant number of cases (approximately 40%–50%) do not have known causes, and these cases are called unexplained recurrent spontaneous abortion (URSA) [3]. It has been proposed that URSA belongs to an alloimmune disease associated with the failure of fetal-maternal immunologic tolerance [4, 5]. Treg cells play a critical role in the induction of a privileged tolerant microenvironment at the fetal-maternal interface [6].

Growing evidence [7–9] suggests that women with URSA had remarkably reduced frequencies of CD4^+^CD25^+^Treg cells in peripheral blood as well as in deciduas. The reduction of Treg cell in URSA patients is closely related to the decreased expression of Foxp3 [9]. Moreover, a reduced suppressive capacity of Treg cells has also been implicated in URSA patients [10]. It is the reduced numbers and/or functional deficiency of CD4^+^CD25^+^Treg cells that cause the predisposition to miscarriage. 

 Foxp3 is a master regulator gene for the development and function of Treg cells [11]. Deficiency of the Foxp3 gene impairs the suppressive function of Treg cells [12]. It has been reported that there are associations between Foxp3 gene polymorphisms and autoimmune diseases, such as systemic lupus erythematosus (SLE) [13], autoimmune thyroid diseases (AITDs) [14], type I diabetes (TID) [15], and allergic rhinitis [16]. However, the association between Foxp3 polymorphisms and URSA has not been defined so far. Thus, the purpose of this study was to determine if functional polymorphisms at the Foxp3 loci were associated with URSA in humans. We will focus on the following four loci of Foxp3 gene: rs2232365A/G, rs5902434del/ATT, rs3761548A/C, and rs2294021T/C. 

## 2. Materials and Methods

### 2.1. Subjects

A total of 146 unrelated URSA patients (age: 22–40 years with a median age of 29.1 years) were selected from the Center of Stem Cell Biology and Tissue Engineering, Sun Yat-sen University (Guangzhou, China). All patients were treated with immunization using paternal lymphocytes during the period between January 2009 and October 2010. All patients had histories of at least two successive miscarriages with unexplained etiology before 12 weeks of gestation, and there was no successful pregnancy record (with the same partner) before this treatment. The median of the number of miscarriages was 3.1. The diagnosis of “unexplained” abortion was made by the following practical guidelines [8]: (1) uterus and cervical abnormalities were excluded by pelvic examination, ultrasound, and a diagnostic hysteroscopy; (2) chlamydia and ureaplasma were excluded by Cervical mucus culturing; (3) chromosome problems were excluded by Karyotypes of abortion couples and abortuses; (4) luteal function defect, hyperprolactinemia, and hyperandrogenemia were excluded by comprehensive hormonal examinations; (5) endocrine diseases, for example, diabetes, hyperthyroidism, and hypothyroidism, were excluded; (6) autoimmune factors associated with systemic lupus erythematosus (SLE) and the antiphospholipid syndrome (APS) such as antinuclear antibodies (ANA), lupus anticoagulant (LA), and anticardiolipin antibodies (ACL) were tested in three consecutive visits every other month; (7) all male partners had normal semen status. The control group containing 112 women (age: 23–44 years old) with at least one live birth was derived from volunteers undergoing routine annual gynecological examination in the same hospital between 2009 and 2010 and there had been no history of spontaneous abortion, preterm labor, or preeclampsia. The subjects in the control group were also examined on the endocrine and immune factors to exclude individuals with diabetes, AITDs, SLE and APS. These stringent criterions described above were used for selection of each subject to exclude borderline cases and enhance the reliability of the data. Genomic DNA was extracted from peripheral blood mononuclear cells using AxyPrep TM blood DNA extraction Mini Kit (AXYGEN, Pittsburgh, Pa, USA) according to the manufacturer's instructions. Written consents of the study were obtained from all patients and the control population after the detailed information sessions with each individual. The study protocol was approved by the Ethics Committee of Sun Yat-Sen University.

### 2.2. Genotyping of rs2232365A/G and rs5902434del/ATT Polymorphisms

Genotypes of rs2232365A/G and rs5902434del/ATT were determined by using the polymerase chain reaction (PCR) with sequence-specific primers (PCR-SSP, Table 1). PCR was performed in a volume of 30 *μ*L, containing 1 *μ*L (20 ng) of genomic DNA, 2 *μ*L (10 pmol) of each primer, 15 *μ*L of Takara Ex Tap Mix and 10 *μ*L of ddH2O. A “touch down” procedure was applied after an initial preheating step for 1 min at 98°C. The PCR parameter for amplification of rs2232365 locus was as follows (the annealing temperatures for rs5902434 was 62°C, 57°C, and 51°C, resp.).

The amplified PCR products were analyzed using 1.5% agarose gel electrophoresis, stained with ethidium bromide and photographed.

### 2.3. Genotyping of rs3761548A/C and rs2294021T/C Polymorphisms

Genotypes of rs3761548A/C and rs2294021T/C were determined using PCR-restriction fragment length polymorphism (PCR-RFLP, Table 1) method. PCR was performed in a volume of 30 *μ*L, with 1 *μ*L (20 ng) of genomic DNA, 2 *μ*L (10 pmol) of each primer, 15 *μ*L of Takara Ex Tap Mix and 10 *μ*L of ddH2O, The parameters for PCR include an initial denaturing step at 98°C for 1 min, followed by 35 cycles of 98°C for 30 sec, annealing for 30 sec, and extension at 72°C for 1 min, and a final extension at 72°C for 7 min. A 15 *μ*L aliquot was digested with 1*μ*L restriction enzyme at 37°C for 16 hours and then separated on a 2% agarose gel. To ensure that the results were repeatable, a 10% sample of the subjects in the patient group and the control group were genotyped twice and the reproducibility was 100%.

### 2.4. Statistical Analysis

Hardy-Weinberg equilibrium (HWE) test, linkage disequilibrium test, and haplotype frequencies for pairs of alleles were estimated using software HAPLOVIEW (version 4.2). The genotype, allele frequency in URSA patients and control group were analyzed by standard Chi-square test. Unconditional univariate and binary logistic regression analyses were performed to obtain the odds ratio (OR) for risk of URSA at 95% confidence intervals (CI). All statistical analysis were performed using the SPSS 13.0 software package (SPSS, Chicago, I/l, USA).

## 3. Results

### 3.1. Identification of Genotypes of the Foxp3 Polymorphisms

**Figure 1:**
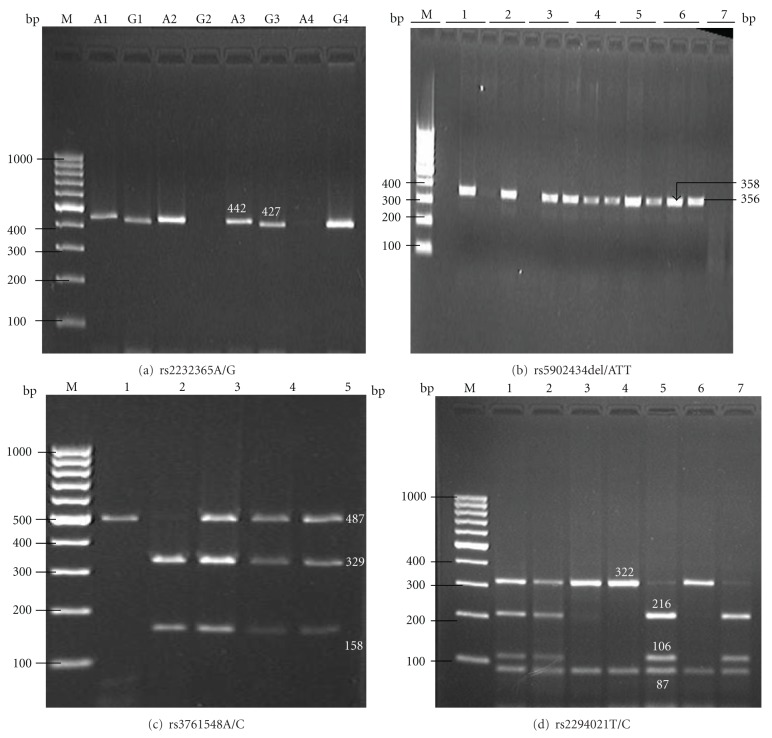
Typical band patterns of PCR-SSP or PCR-RFLP products. (a) is the band pattern for rs2232365. Lane 1 and 3 indicated A/G genotype; Lane 2 indicated A/A genotype; Lane 4 indicated G/G genotype. (b) is the band pattern for rs5902434. Lane 1, 2, and 3 indicated ATT/ATT genotype; Lane 4, 5, and 6 indicated del/ATT genotype; Lane 7 indicated del/del genotype. (c) is the band pattern for rs3761548. Lane 1 indicated A/A genotype; Lane 2 indicated C/C genotype; Lane 3, 4, and 5 indicated A/C genotype. (d) is the band pattern for rs2294021. Lane 1, 2 indicated T/C genotype; Lane 3, 4, and 6 indicated C/C genotype; Lane 5, 7 indicated T/T genotype. M represented 100 bp DNA marker.

For genotyping of rs2232365A/G and rs5902434del/ATT, each DNA sample was tested in duplicates with one reaction per allele of each locus. The A/A, A/G, and G/G genotypes had one (442 bp), two (442 bp and 427 bp), and one (427 bp) band(s), respectively. The del/del, del/ATT, and ATT/ATT genotypes also had one (358 bp), two (358 bp and 356 bp), one (356 bp) band(s), respectively. Genotypes of rs3761548A/C were defined by the presence of three different bands: A/A (487 bp), A/C (487 bp, 329 bp and 158 bp), and C/C (329 bp, 158 bp). For rs2294021T/C, genotypes were defined by the presence of five distinct patterns of bands: T/T (322 bp, 87 bp, and 20 bp), T/C (422 bp, 216 bp, 106 bp, 87 bp, and 20 bp), C/C (216 bp, 106 bp, 87 bp, and 20 bp). The 20 bp band was running out of the gel. The representative gels showing the typical patterns of bands were presented in Figure 1.

### 3.2. Genotypic and Allelic Distribution of the Foxp3 Polymorphisms in the Subjects of URSA and Control Groups

**Figure 2:**
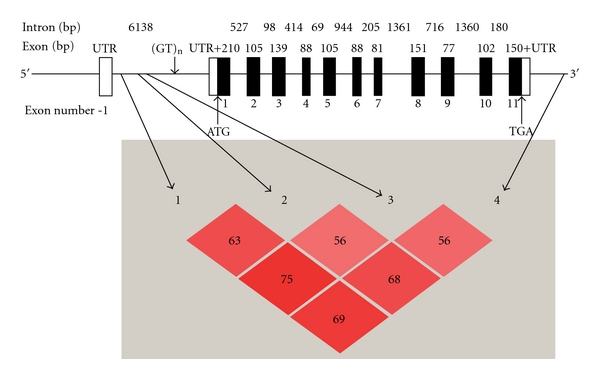
Schematic structure of the human Foxp3 gene and the linkage disequilibrium as determined in the URSA population (Haploview 4.20 version). The coding sequences, noncoding sequences and the introns were depicted as black boxes, white boxes, and horizontal lines, respectively. Exon numbers were shown below the exon boxes. The sizes of exons and introns were labeled above them. The locations of rs5902434 (1), rs3761548 (2), rs2232365 (3), rs2294021 (4), (GT)n microsatellite polymorphisms, the start cordon, and the stop cordon were indicated by arrows. The standard (D/LOD) was represented by red color and *r* ^2^ was presented as a number if it deviates from 100.

All the four SNPs were in HWE for both URSA group and control group with a *P* value from 0.08 to 0.85. In addition, these 4 SNPs were under linkage disequilibrium in the URSA group (Figure 2). The genotypic and allelic frequencies data were shown in Table 2. We performed statistical analysis to determine the association between each of the SNPs and URSA. There was no statistical difference between the genotypic and allelic frequencies of rs2294021T/C polymorphism in the URSA group and those in the control group. The genotypic frequencies of rs5902434 del/ATT polymorphism did not differ significantly between the URSA and control groups, but a weak association was observed in the allelic distribution (*P* = 0.036) between URSA and control groups. The genotypic and allelic distributions of rs2232365 A/G and rs3761548A/C were statistically different between the URSA and control groups. Consistent with the rs2232365 G allelic distribution, the frequency of the combined genotypes (A/G + G/G) in the URSA group (94.5%) was significantly higher than that in the control group (84.82%) (*P* = 0.009). The risk of URSA in women with G allele was higher than that in women with A allele (OR = 1.61, 95% CI = (1.11, 2.32)). Similar to the locus of rs2232365, the distribution of genotypes and alleles at the locus rs3761548 was also significantly different between the URSA and control groups.

### 3.3. Analysis of Haplotypes Based on the Three Polymorphisms in the Promoter Region

Because haplotypes are clusters of genetic variants and are inherited as a unit on the same chromosome, we stratified the subjects in URSA group and control group based on the Foxp3 promoter region haplotypes to explore the relative influence of each individual haplotype on URSA susceptibility. Of the 8 haplotypes (Table 3) defined by three polymorphisms at positions rs5902434, rs3761548, and rs2232365, they were classified into four distinct groups: ATT-C-A, ATT-A-A, del-A-G and del-C-G. The frequencies of the latter 3 haplotypes (ATT-A-A, del-A-G, and del-C-G) were significantly different between URSA and control groups. The proportion of haplotype ATT-A-A and Del-C-G in URSA group was significantly lower than that in the control group, indicating that these haplotypes play a protective role in the occurrence of URSA, In contrast, haplotype del-A-G is a risk factor of URSA (OR = 2.51.95% CI = (1.75, 3.58)).

## 4. Discussion

Treg cells play a central role in the induction and maintenance of fetal-maternal immunologic tolerance. Development of Treg cells requires continued expression of Foxp3 [17], while attenuated Foxp3 expression results in its functional deficiency [18], We hypothesized that functional polymorphisms of the Foxp3 gene may contribute to the pathogenesis of URSA. 

In the cases of the rs3761548A/C polymorphism, the A allele was a risk factor for SLE [13], AITDs [14], allergic rhinitis [16]. Interestingly, we found that genotypic frequency of rs3761548AA was significantly different between the URSA group and control group. Previous studies have shown that individuals with a genotype of rs3761548AA have the lowest production of Foxp3 among the three genotypes of this polymorphism [19]. Therefore, URSA patients with the AA genotype may have fewer Treg cells and/or weaker suppressive function and are difficult to achieve fetal tolerance, these results are consistent with previous reports, showing a decrease in the proportion of Foxp3+ Treg cells in URSA patients [8, 9]. Taken together, our results suggested that the rs3761548AA genotype may contribute to the occurrence of URSA.

For the polymorphism of rs2232365A/G, the distribution of alleles and genotypes differ significantly between the URSA group and control group. The risk of URSA in the women with G allele was higher than that in the women carrying an A allele (OR = 1.61, 95% CI = (1.11, 2.32)). These results are also consistent with the previous work [15], showing that Type I diabetes (TID) carrying a G allele has a higher occurrence of beta-cell failure. Because the functional differences between different genotypes are still unclear, we performed an extensive search for transcriptional factor-binding sites using TF-Search online tool (http://www.cbrc.jp/research/db/TFSEARCH.html). Interestingly, SNP variant of rs2232365A/G is located in a putative binding site for the transcription factor GATA-3, known to be essential for the Th2 immune response. More importantly, only when the A allele exists, can GATA-3 bind the promoter region of Foxp3. Th1/Th2 cytokine balance with Th2 predominance is a very important mechanism for the survival of the fetus in the maternal uterus [20]. It is also shown that Treg cells modulate the Th1/Th2 cell balance toward Th2 cell, and thus upregulate Th2 immune response [21]. Every recently, an intrinsic mechanism predisposing Foxp3-expressing regulatory T cells to Th2 conversion through GATA-3 in vivo has been identified [22]. Hence, we inferred that high frequencies of G allele and G/G genotype in URSA patients might decrease Th2 immune response and disrupt the balance of Th1/Th2, leading to a detrimental effect on the fetus during pregnancy

The human Foxp3 gene is located in the small arm of the X-chromosome. Random inactivation of X-chromosome results in the presence of only two phenotypes for each polymorphism [23], because the phenotype of the heterozygous genotype is identical to that of a homozygous genotype. Therefore, we performed stratification analysis based on the possible phenotypes. Stratification of the URSA patients and controls by heterozygotes and homozygotes failed to detect any evidence that the heterozygotes were more frequent in URSA group, which was in contrast to the results from the previous studies [14, 16]. Among the 4 SNPs, however, the combinational genotypes del/del + del/ATT, A/G + G/G, C/C + A/C, and A/G + G/G appeared significantly different between the URSA and control groups, whereas only A/G + G/G and C/C + A/C were still significant after the Bonferroni correction for multiple testing of three tests(*P*≦0.017).These data, combined with the results from the haplotype analysis further provided evidence for the association between Foxp3 gene and URSA and supported that immune dysregulation is secondary to promoter heterogeneity in the Foxp3 gene. While some correlation has been found in the study, it is still very important to examine a larger number of samples from different populations and to investigate the proportions of Treg cells and Foxp3 expression or even their suppressive capacity in peripheral or decidua among different genotypes in URSA patients to obtain more reliable conclusions.

## 5. Conclusions

We have evaluated the role of Foxp3 gene functional polymorphisms in the pathogenesis of URSA. Two functional polymorphisms in Foxp3 gene (rs2232365A/G and rs3761548A/C) are related to the occurrence of URSA. These results highlight the important role of Foxp3 in successful pregnancy. 

##  Conflict of Interests

The authors have no financial conflict of interests.

## Figures and Tables

**Figure 3:**
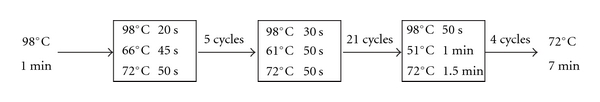


**Table 1:** Primers used in the genotyping by PCR-SSP or PCR-RFLP.

Marker	Allele	Forward primer	Reverse primer	PCR product Tm/°C
Method	Enzyme			
rs2232365	A	5′-CCCAGCTCAAG	5′-GGGCTAGTGAG	442 bp
PCR-SSP		AGACCCCA-3′	GAGGCTATTGTAA C-3′	
	G	5′-CCAGCTCAAGA	5′-GCTATTGTAACA	427 bp
		GACCCCG-3′	GTCCTGGCAAGTG-3′	
rs5902434	deletion	5′-ACCTTTAAGTCTTCTGCC	5′-TGATTATCAGCG	356 bp
PCR-SSP		ATTTATTCTATTATTT-3′	CACACACTCAT-3′	
	ATT	5′-CCTTTAAGTCTTCTGCCA	5′-TGATTATCAGCG	358 bp
		TTTATTCTATTATTA-3′	CACACACTCAT-3′	
rs3761548	PstI	5′-GCCCTTGTCTA	5′-CAGCCTTCGCCA	487 bp
PCR-RFLP		CTCCACGCCTCT-3′	ATACAGAGCC-3′	63°C
rs2294021	HaeIII	5′-CACACACAATCCAT	5′-ATCTCCATGCCCTA	429 bp
PCR-RFLP		CCCAGTCACCC-3′	AGAAGGCCACC-3′	59°C

**Table 2:** Genotype and allele frequencies of the four Foxp3 polymorphisms in controls and URSA patients together with case-control analysis.

Marker	Genotype allele	URSA *N* (%)	Controls *N* (%)	*χ* ^2^ value	*P* value	OR (95% CI)
rs2294021	C/C	65 (44.50)	40 (35.70)			
	T/C	70 (47.90)	57 (50.90)			
	T/T	11 (7.60)	15 (13.40)	3.480	0.18^a^	
	C/C	65 (44.50)	40 (35.70)			
	T/C + T/T	81 (55.50)	72 (64.30)	2.037	0.154^b^	
	T/C	70 (47.90)	57 (50.09)			
	C/C + T/T	76 (52.10)	55 (49.10)	0.220	0.639^b^	
	T/T	11 (7.60)	15 (13.40)			
	C/C + T/C	135 (92.40)	97 (86.60)	2.401	0.121^b^	
rs5902434	ATT/ATT	11 (7.53)	14 (12.50)			
	del/ATT	62 (42.47)	56 (50.00)			
	del/del	73 (50.0)	42 (37.50)	4.620	0.10^a^	
	ATT/ATT	11 (7.53)	14 (12.50)			
	del/ATT + del/del	135 (92.47)	98 (87.50)	1.786	0.181^b^	
	del/ATT	62 (42.47)	56 (50.00)			
	del/del + ATT/ATT	84 (57.53)	56 (50.00)	1.450	0.229^b^	
	del/del	73 (50.0)	42 (37.50)			
	del/ATT + ATT/ATT	73 (50.00)	70 (62.50)	4.008	0.045^b^	0.60 (0.36, 0.99)
rs2232365	A/A	8 (5.48)	17 (15.18)	8.320	0.016^a^	1
	A/G	70 (47.94)	56 (50.00)	4.640	0.031^b^	2.66 (1.07, 6.61)
	G/G	68 (46.58)	39 (34.82)	8.260	0.004^b^	3.71 (1.47, 9.37)
	A/G	70 (47.94)	56 (50.00)			
	A/A + G/G	76 (52.06)	56 (50.00)	0.107	0.743^b^	
	G/G	68 (46.58)	39 (34.82)			
	A/A + A/G	78 (53.42)	73 (65.18)	3.607	0.058^b^	
	A/A	8 (5.48)	17 (15.18)			
	A/G + G/G	138 (94.52)	95 (84.82)	6.810	0.009^b^	3.09 (1.28, 7.44)
rs3761548	C/C	15 (10.27)	25 (22.32)	8.280	0.016^a^	1
	A/C	56 (38.36)	45 (40.18)	1.690	0.193^b^	1.44 (0.83, 2.47)
	A/A	75 (51.37)	42 (37.50)	8.620	0.003^b^	2.98 (1.42, 6.26)
	A/C	56 (38.36)	45 (40.18)			
	C/C + A/A	90 (61.64)	67 (59.82)	0.088	0.076^a^	
	A/A	75 (51.37)	42 (37.50)			
	C/C + A/C	71 (48.63)	70 (62.50)	4.920	0.027^b^	0.57 (0.34, 0.94)
	C/C	15 (10.27)	25 (22.32)			
	A/C + A/A	131 (89.73)	87 (77.68)	7.020	0.008^b^	2.51 (1.25, 5.03)
rs2294021	C	200 (68.50)	137 (61.20)			
	T	92 (31.50)	87 (38.80)	3.01	0.080^c^	1.38 (0.96, 1.99)
rs5902434	ATT	84 (28.77)	84 (37.50)			
	del	208 (71.23)	140 (62.50)	4.40	0.036^c^	1.49 (1.03, 2.15)
rs2232365	A	86 (29.45)	90 (40.18)			
	G	206 (70.55)	134 (59.82)	6.50	0.010^c^	1.61 (1.11, 2.32)
rs3761548	C	86 (29.45)	95 (42.40)			
	A	206 (70.55)	129 (57.60)	8.37	0.003^c^	1.73 (1.20, 2.50)

*P* ^a^ was determined by Pearson's Chi-square test for 3 × 2 contingency tables.

*P* ^b^, *P* ^c^ was determined by Pearson's Chi-square test for 2 × 2 contingency tables.

*P* ^a^  ≦0.05,  *P* ^b^≦0.017, *P* ^c^≦0.05 are considered statistically significant.

**Table 3:** Eight three-locus Foxp3 promoter region haplotypes identified in URSA group and the control group and their estimated frequencies using the software HAPLOVIEW (version 4.2).

Allele			Haplotype frequency (*N*, %)				
rs5902434	rs3761548	rs2232365	URSA	Control	*χ* ^2^ value	*P*	OR (95% CI)
del	C	A	6 (2.1)	8 (3.4)	0.824	0.36	
del	A	G	187 (64.1)	93 (41.6)	25.86	0.000	2.51 (1.75.3.58)
del	C	G	9 ( 3.2)	35 (15.8)	25.39	0.000	0.18 (0.08, 0.37)
del	A	A	5 (1.8)	4 (1.7)	0.013	0.91	
ATT	A	G	4 (1.3)	2 (0.7)	0.387	0.53	
ATT	A	A	10 (3.3)	30 (13.5)	18.6	0.000	0.22 (0.10, 0.46)
ATT	C	G	6 (1.9)	4 (1.6)	0.05	0.82	
ATT	C	A	65 (22.3)	48 (21.6)	0.033	0.86	
